# *COMT* Val^158^Met Genotype Determines the Direction of Cognitive Effects Produced by Catechol-*O*-Methyltransferase Inhibition

**DOI:** 10.1016/j.biopsych.2011.12.023

**Published:** 2012-03-15

**Authors:** Sarah M. Farrell, Elizabeth M. Tunbridge, Sven Braeutigam, Paul J. Harrison

**Affiliations:** a Department of Psychiatry, University of Oxford, Warneford Hospital, Oxford, United Kingdom; b Oxford Centre for Human Brain Activity, University of Oxford, Warneford Hospital, Oxford, United Kingdom

**Keywords:** Catechol-*o*-methyltransferase, decision making, pharmacogenetics, polymorphism, tolcapone, working memory

## Abstract

**Background:**

Catechol-*O*-methyltransferase (COMT) metabolizes dopamine. The *COMT* Val^158^Met polymorphism influences its activity, and multiple neural correlates of this genotype on dopaminergic phenotypes, especially working memory, have been reported. COMT activity can also be regulated pharmacologically by COMT inhibitors. The inverted-U relationship between cortical dopamine signaling and working memory predicts that the effects of COMT inhibition will differ according to *COMT* genotype.

**Methods:**

Thirty-four *COMT* Met^158^Met (Met-COMT) and 33 *COMT* Val^158^Val (Val-COMT) men were given a single 200-mg dose of the brain-penetrant COMT inhibitor tolcapone or placebo in a randomized, double-blind, between-subjects design. They completed the N-back task of working memory and a gambling task.

**Results:**

In the placebo group, Met-COMT subjects outperformed Val-COMT subjects on the 2- back, and they were more risk averse. Tolcapone had opposite effects in the two genotype groups: it worsened N-back performance in Met-COMT subjects but enhanced it in Val-COMT subjects. Tolcapone made Met-COMT subjects less risk averse but Val-COMT subjects more so. In both tasks, tolcapone reversed the baseline genotype differences.

**Conclusions:**

Depending on genotype, COMT inhibition can enhance or impair working memory and increase or decrease risky decision making. To our knowledge, the data are the clearest demonstration to date that the direction of effect of a drug can be influenced by a polymorphism in its target gene. The results support the inverted-U model of dopamine function. The findings are of translational relevance, because COMT inhibitors are used in the adjunctive treatment of Parkinson's disease and are under evaluation in schizophrenia and other disorders.

An inverted-U relationship has been proposed between dopamine and prefrontal cortex function in which too little or too much dopamine signaling impairs working memory (1–4). Both genetic and pharmacologic factors can affect position on the curve. The enzyme catechol-*O*-methyltransferase (COMT) metabolizes dopamine and is one such influence (5–9). COMT activity is genetically influenced, with the greatest variance explained by a common polymorphism, Val^158^Met, which is associated with a approximately 35% enzyme activity difference between homozygotes (Val-COMT > Met-COMT) in human brain (10); this is reflected in a genotype difference in cortical D1 dopamine receptor availability, a proxy measure of cortical dopamine (11). In turn, as shown initially by Egan *et al.* (12) and confirmed in many other studies (13), there are robust Val^158^Met genotype effects on cortical activation during tasks of working memory and executive function. Val^158^Met genotype differences in working memory performance have also been reported (12,14), although less consistently (15). COMT activity can also be regulated pharmacologically by COMT inhibitors, with corresponding cognitive, behavioral, and neurochemical effects, in rodents (16,17) and humans (18–21).

The inverted-U model predicts that COMT inhibition should have differential effects on working memory depending on Val^158^Met genotype. That is, the effect of being moved rightward on the curve (because COMT inhibition increases prefrontal dopamine) (16) will be affected by the starting position: Val-COMT subjects, with their higher COMT activity, sit to the left of Met-COMT subjects. COMT inhibition will thus tend to move Val-COMT subjects closer to the optimum and enhance performance, whilst moving Met-COMT subjects beyond the peak and impairing performance. To date, tests of this focused pharmacogenetic hypothesis are intriguing but inconclusive (19,20), although *COMT* Val^158^Met genotype has been shown to modulate responses to other dopaminergic drugs including amphetamine (22), antipsychotics (23), and methylphenidate (24).

Here we recruited Val-COMT and Met-COMT homozygote men, gave them the brain-penetrant COMT inhibitor tolcapone (25,26), or placebo, and measured their performance on the N-back task of working memory. Because the possibility that an inverted-U relationship may extend to other dopamine-modulated phenotypes remains less well explored, we also tested the subjects' performance on a gambling task.

## Methods and Materials

### Participants

The study was approved by the Oxfordshire National Health Service Research Ethics Committee B (09/H0605/69). Healthy men aged 18 to 50 years old were recruited by advertisement. They had no history of psychiatric or neurologic disorder, and none were taking psychotropic medication. Alcohol and smoking use was recorded, and all subjects denied use of illicit substances. Subjects with alcohol intake greater than 30 units/week or a history of liver disease were excluded because of the hepatotoxicity risk with tolcapone. Participants were genotyped for the Val^158^Met polymorphism. We selected only homozygotes (Met-COMT and Val-COMT), because these represent low and high COMT activity, respectively, with heterozygotes being intermediate (10) and therefore less informative in the present context. The subjects were unrelated to each other. Sixty-seven subjects performed the gambling task, 60 of whom also carried out the N-back (Table 1). Subjects completed the National Adult Reading Test and depression and anxiety inventories. On the day of testing, they completed visual analogue scale (VAS) ratings of alertness, drowsiness, happiness, sadness, anxiety, and nausea; these were completed on arrival and again approximately 90 min and then approximately 120 min later.

Within each genotype group, subjects were randomly assigned to tolcapone (200 mg by mouth) or placebo. A between-subjects design was chosen to avoid order or practice effects. The study was double-blind, with matching capsules prepared by a pharmacy registered under U.K. Good Manufacturing Practice regulations. Tolcapone has an elimination half-life of 2.0 ± .8 hours, and the dose given produces 70% to 80% peripheral blood COMT inhibition between 1 and 4 hours (25,27). Testing began 90 min after swallowing the capsule and lasted ∼90 min. Testing was carried out while subjects were in a magnetoencephalography (MEG) scanner; MEG results will be reported separately. After testing, subjects were asked to guess whether they had received tolcapone or placebo.

### Genotyping

DNA was extracted from buccal swabs and genotyped for Val^158^Met using the appropriate Taqman SNP Genotyping Assay (Applied Biosystems, Carlsbad, California), in duplicate. A subset of genotypes were confirmed using a restriction fragment length polymorphism assay (28). Genotype calls agreed in all cases. No other polymorphisms were measured, reflecting the hypothesis-driven nature of the study.

### N-Back Task of Working Memory

In the N-back task as instantiated here, a number between 1 and 4 is shown at random on a screen. For the 0-back, subjects respond to the number currently showing on the screen by pressing the appropriate button; for the 1-back, subjects respond to the previous number on the screen, and so on for 2- and 3-back conditions. Each number is shown for 160 msec, with an interval of 1640 msec between numbers (and 3000 msec between blocks). The task imposes a parametric load on working memory, and the version we used is relatively demanding (29). The primary performance measure is accuracy (correct responses); we also measured reaction time (RT).

### Gambling Task

The task involves monetary decision making based on a choice between gambling a high or a low amount (30). The task has been used extensively for studies of decision making and risky choice; electroencephalographic responses during the task are sensitive to *COMT* Val^158^Met (31). Subjects are given £10 (approximately $15) to play with. Two gray boxes are shown on a screen, with “5” or “25” shown within each box. The subject selects one box, and the choice means they are gambling either 5 or 25 pence. Once the choice is made, the boxes change color, either to green (indicating a win) or to red (indicating a loss). Both boxes may go red, both green, or one of each. Thus, a subject can win when they could have lost, lose when they could have won, win a small amount when they could have won a large amount, and so on. Intermittently, the screen shows how much money is currently banked. The primary measure of interest is simply the percentage of bets that were “5” not “25.” We also calculated this for bets that followed two successive wins, or two successive losses, because Val^158^Met genotype has been reported to affect sensitivity to losses versus rewards (32,33).

### Statistical Analysis

Analyses were carried out in SPSS for Windows (version 17.0; SPSS Statistics Inc., Chicago, Illinois). For the N-back, we first conducted a repeated-measures analysis of variance (ANOVA), with difficulty (back condition) as the within-subjects factor and drug and genotype as the between-subjects factors. Each back condition was then examined with a two-way ANOVA with drug and genotype as between-subjects factors. Post hoc comparisons were made using *t* tests (two-tailed). For the gambling task, we used two-way ANOVA with drug and genotype as between-subjects factors. Correlations between variables were explored using Pearson's coefficient. Significance was set at α = .05.

## Results

The drug blind was maintained, with 59 of the 67 subjects thinking that they had received placebo, including 30 of the 33 who had in fact had tolcapone.

### N-Back Performance

**Figure 1:**
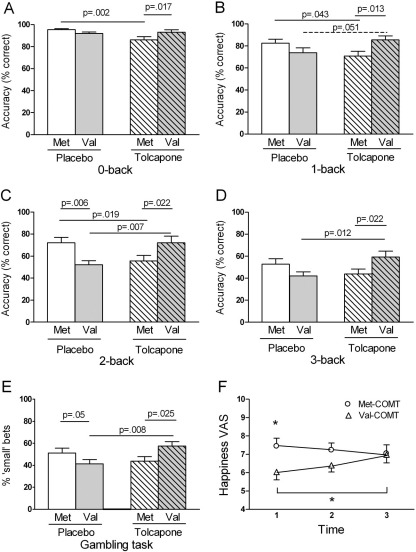
Interactive behavioral effects of catechol-*O*-methyltransferase (*COMT*) genotype and COMT inhibition. **(A)** 0-back: main effect of drug [*F*(1,56) = 4.14, *p =* .047] and genotype × drug interaction [*F*(1,56) = 6.75, *p =* .012]. Met-COMT subjects given tolcapone perform worse than those given placebo. **(B)** 1-back: genotype × drug interaction [*F*(1,56) = 9.26, *p =* .006]. Tolcapone impairs performance in Met-COMT subjects and, as a trend, improves it in Val-COMT subjects, compared with their respective placebo groups. **(C)** 2-back: genotype × drug interaction [*F*(1,56) = 13.62, *p =* .001]. Met-COMT subjects perform better than Val-COMT subjects on placebo. Tolcapone reverses this difference, impairing Met-COMT subjects and enhancing Val-COMT subjects, compared with their respective placebo groups. **(D)** 3-back. Genotype × drug interaction [*F*(1,56) = 8.03, *p =* .006]. Val-COMT subjects given tolcapone perform better than those given placebo. **(E)** Gambling task, showing percentage of times when 5 not 25 was gambled. Genotype × drug interaction [*F*(1,61) = 7.91, *p =* .007]. On placebo, Met-COMT subjects are more likely than Val-COMT subjects to make a small rather than a large bet. Tolcapone reverses this difference, making Val-COMT subjects significantly more risk averse, compared with those given placebo. **(F)** Happiness visual analogue scale (VAS) ratings for tolcapone-treated subjects. Data are adjusted means with standard errors. Time 1: immediately before drug administration. Time 2: T1 + 90 min. 3: T1 + 210 minutes. *p = .026. See Table S2 in Supplement 1 for happiness VAS ratings in the placebo groups.

There were no main effects of drug or genotype on N-back accuracy, nor interactions between drug or genotype and difficulty. There was a genotype × drug × difficulty interaction from 0-back to 3-back [*F*(2.33,130.3) = 3.9, *p* = .018], and a genotype × drug interaction for all conditions (Figure 1 and Figure S1A in Supplement 1).

The most striking results were seen for the 2-back (Figure 1C). On placebo, Met-COMT subjects out-performed Val-COMT subjects (*p =* .006), but in the tolcapone group, this difference was reversed such that Val-COMT subjects outperformed Met-COMT subjects (*p =* .022); tolcapone significantly impaired performance in Met-COMT subjects (*p =* .019) and improved it in Val-COMT subjects (*p =* .007). For the 0- and 1-back, on placebo, Met-COMT and Val-COMT subjects performed similarly, but accuracy was impaired in Met-COMT subjects given tolcapone compared with those given placebo (0-back, *p =* .002; 1-back, *p =* .043; Figure 1A and 1B). Consequently, in subjects given tolcapone, Val-COMT subjects performed better than Met-COMT subjects (0-back, *p =* .017; 1-back, *p =* .013). Results for the 3-back were similar to the 2-back but less significant (Figure 1D). The genotype × drug interaction accounted for 19% of overall N-back performance (partial h^2^), and for 13.5%, 9%, 15.5%, and 9% of the variance in 0, 1, 2, and 3-back performance, respectively (adjusted *R*^2^).

Because a significant drug × genotype interaction was seen for the 0-back condition, in which working memory is not explicitly required, we performed a supplementary analysis to investigate whether COMT affects working memory when 0-back performance is controlled for. For each subject, 1-, 2-, and 3-back performance was expressed as a percentage of 0-back performance and entered into a repeated-measures ANOVA with three levels of difficulty (1-, 2-, and 3-back). This analysis showed a drug × genotype interaction [*F*(1,60) = 9.2; *p =* .004], with Met-COMT subjects outperforming Val-COMT subjects in the placebo groups (*p =* .024) and Val-COMT subjects tending to outperform Met-COMT subjects in the tolcapone groups (*p =* .053). Thus, an interaction between drug and genotype on working memory remained when 0-back performance was taken into account.

Reaction times for each N-back condition are shown in Table 2 and Figure S1B in the Supplement. We found a trend-level RT × genotype interaction [*F*(1.86,98.7) = 2.79, *p =* .070], with Met-COMT subjects reacting faster than Val-COMT subjects (*p =* .049). Tolcapone decreased RT in Val-COMT (*p =* .046) but not in Met-COMT (*p =* .61) subjects. On placebo, Met-COMT subjects reacted faster than Val-COMT subjects on the 2-back (*p =* .046) and 3-back (*p =* .039); no genotype differences in RT were seen for subjects on tolcapone.

### Gambling Task

There was a genotype × drug interaction for the size of bet placed [*F*(1,61) = 7.91, *p =* .007]. In the subjects given placebo, Met-COMT subjects made more small bets than Val-COMT subjects (*p =* .05); this difference was reversed in subjects given tolcapone (*p =* .025; Figure 1E), with tolcapone-treated Val-COMT subjects being more risk averse than tolcapone-treated Met-COMT subjects (*p =* .008). Drug and genotype together explained 8.5% of the variance. As noted, Val^158^Met genotype may affect sensitivity to losses versus rewards (32,33), but our findings were unaffected by the outcomes of prior trials. That is, the main effects and interactions shown in Figure 1E were also seen for trials following two wins or two losses or other combinations of prior outcomes (Table 2 and data not shown). We found no main effects of drug or genotype, nor interactions, on gambling task RT (Table 2; all *F*s <1.5, *p* > .3).

### Other Measures and Correlations

On the VAS ratings, Met-COMT subjects rated themselves happier than Val-COMT subjects overall [7.2 ± .3 vs. 6.3 ± .3; *F*(1,61) = 5.58, *p =* .021], including at baseline [7.1 ± .3 vs. 6.0 ± .3; *p =* .013]. Happiness also showed a drug × genotype × time interaction [*F*(1.70,103.9) = 3.3, *p =* .037] due to an increase in ratings with time in the Val-COMT tolcapone group (*p =* .015, comparing time points 1 and 3) that was absent in other groups (Figure 1F). None of the other VAS ratings were affected by genotype or drug (Table S1 in Supplement 1), nor did the groups differ in their depression and anxiety inventory ratings (Table 1).

Because we found drug × genotype interactions on N-back performance, risky decision making, and the happiness VAS rating, we investigated whether these variables correlated with each other. However, there were no significant correlations (−.13 < R < .23; all *p*s > .05; Table S2 in Supplement 1). Neither did inclusion of happiness VAS score as a covariate affect the N-back or gambling task results (data not shown).

## Discussion

Our study has two main findings. First, on placebo, Met-COMT subjects outperformed Val-COMT on the 2-back task of working memory and were more risk averse. Second, and more notably, genotype interacted with COMT inhibition by tolcapone to affect both these indices. Compared with those given placebo, tolcapone improved working memory in Val-COMT subjects but impaired it in Met-COMT subjects; it made Met-COMT subjects less risk averse and Val-COMT subjects more so. These interactions were qualitative and robust, and the drug effect was sufficient to reverse baseline genotype differences. The effects were also of notable size, with Val^158^Met genotype and tolcapone together accounting for 19% of the variance in N-back performance. This is substantial, especially given that we did not include the contribution of other genetic (21,34,35) and epigenetic (36) sources of COMT variation nor polymorphisms in interacting dopaminergic genes (37–39). These results are significant with regard to the range of phenotypes with which COMT is associated, the inverted-U model of cortical dopamine, and from a pharmacogenetic perspective. Our findings support the hypothesis that *COMT* genotype influences not just the magnitude but the direction of cognitive and behavioral responses to COMT inhibition.

Our findings that, on placebo, Met-COMT subjects performed better than Val-COMT subjects at the 2-back replicates the result of one large study (15,40) but many other studies (e.g., Blanchard *et al.*) (41) and a meta-analysis (14) have been negative. The differences likely reflect the fact that the nature of the N-back task differs between different versions; we used one with high updating and interference management demands thought to be more dependent on COMT (29,42). Our task was also relatively difficult, as shown by the performance data, perhaps because subjects were new to the task and given only brief instruction, and they completed it in the MEG scanner. Limiting our study to young men with an above-average and restricted range of verbal IQ (National Adult Reading Test scores ranged from 108 to 125) may also have contributed, given gender- (43,44), age- (45,46), and possibly IQ-related (14) variation in COMT function.

Two prior studies have investigated interactive effects of tolcapone and Val^158^Met on working memory and executive functioning and gave more equivocal results than we report here. Apud and colleagues (19) used repeated tolcapone administration (200 mg three times a day for a week) in a within-subjects crossover design. They did not identify any main effects or interactions on N-back accuracy but did find an interaction on intradimensional set shifting, such that tolcapone improved Val-COMT subjects but impaired Met-COMT subjects. Giakoumaki *et al.* (20) used a single dose of tolcapone (200 mg) in a within-subjects crossover design. They found no drug × genotype interaction for N-back accuracy but did for N-back RT, which was improved by tolcapone in Val- but not Met-COMT subjects. They also found a trend-level effect on letter-number sequencing, another test of working memory, with tolcapone again selectively improving Val-COMT subjects. Overall, therefore, all studies agree that tolcapone and genotype have interactive effects on executive functioning, with tolcapone enhancing Val-COMT subjects but either not improving or impairing Met-COMT subjects. The demographic and methodological factors mentioned earlier are probably relevant in explaining why we identified more robust drug × genotype interactions, specifically on N-back performance, than did the prior studies; moreover, our sample of homozygotes (*n* = 60–67) is considerably larger than the number included in the earlier studies (*n* = 22–23).

Our other findings highlight that COMT has roles in a range of phenotypes beyond executive function and working memory (6,7). First, involvement of COMT in dopaminergic contributions to decision making and reward has hitherto been demonstrated in relatively complex paradigms (47–50), but our gambling task data show that even the simple trait of risky decision making is affected by COMT: high COMT activity is associated with making a higher proportion of large gambles (risky choices) compared with low COMT activity. This finding may relate to the recent demonstration in rodents that enhanced dopamine signaling via D2 receptors promotes risk aversion (51). Second, the effect of tolcapone on 0-back performance suggests an influence of COMT on attention, consistent with prior evidence for COMT modulation of attentional processes (52,53). Third, we found some evidence for an effect on mood state (the happiness VAS), which may relate to evidence linking *COMT* Val^158^Met with affective processing (12,54). However, none of our other trait or state ratings nor the earlier tolcapone studies (19,20) found any effects of tolcapone on affect or well-being, and so we view this result with caution. Nevertheless, the results in total emphasize the pleiotropic nature of COMT and the need to consider affective and attentional contributions to its modulation of working memory or other higher level cognitive processes.

**Figure 2:**
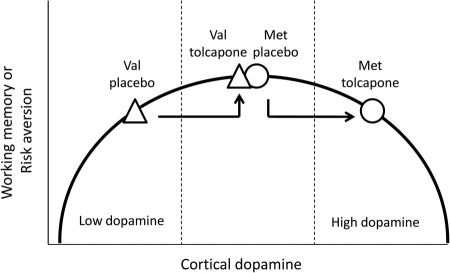
Catechol-*O*-methyltransferase (*COMT*) genotype, inhibition, and the dopaminergic inverted-U. Val-COMT subjects have higher COMT activity and thus lower dopamine tone than Met-COMT subjects, and therefore sit further to the left on the curve. After tolcapone, all subjects move to the right because dopamine signaling increases. However, the functional correlates of this shift differ between genotypes. On the N-back task, Val-COMT subjects move closer to the optimum, whereas Met-COMT subjects are now to the right of the peak. A similar principle applies to the gambling task, although in this case, the y axis is not performance but risk aversion.

The genotype × drug interactions support the inverted-U model of dopamine function, and for a genetic and pharmacologic contribution from COMT to this relationship, based on the rationale outlined earlier and illustrated in Figure 2. The gambling task data highlight that an inverted-U may well apply not only to working memory but to other dopamine-modulated behaviors (2,4). It is possible that the various findings could be explained in terms of different manifestations of a single inverted-U, but the absence of any correlations between COMT's effects on working memory and risky decision making suggests that there is at least a partial separation of the underlying neural circuits and mechanisms. However, in humans, it is difficult to test critically the inverted-U concept or to distinguish between variants of it. Instead, formal testing can better be conducted in pharmacologic studies of humanized transgenic mice that mimic the human Val^158^Met polymorphism (55). Such studies will also provide the opportunity to investigate the cellular and synaptic processes involved. For example, the balance between D1 and D2 receptor signaling, high and low D2 receptor affinity states, and other putative mechanisms (8).

In summary, our results provide a striking and “lawful” example of a genotype × drug interaction. That is, the functionality of the gene is known (viz., it metabolizes dopamine), as is that of the Val^158^Met polymorphism (viz., it influences enzyme activity); the drug effectively and selectively targets the protein encoded by the gene; and the predicted effects are grounded in a well-established systems-level model (viz., the inverted-U of cortical dopamine signaling). Such a pharmacogenetically favorable set of circumstances is rare and may explain why, to our knowledge, COMT provides the clearest demonstration to date of a qualitative pharmacogenetic effect in which not just the magnitude but the direction of the behavioral effect of a drug is determined by variation in sequence of its target gene. Equally, the fact that prior studies did not find such clear cut results attests to the many other factors that can obscure this relationship.

Finally, the findings have translational implications. COMT inhibitors are licensed as adjunctive therapy for Parkinson's disease and are under investigation in schizophrenia and other neuropsychiatric conditions (56,57). Extending earlier findings (19,20), our results show that *COMT* Val^158^Met genotype can affect the cognitive, behavioral, and perhaps affective responses to these drugs; genotype may also influence their toxicity (58). Thus, genotype will be worth measuring in the ongoing trials and including in the design of future ones. However, the results will likely vary between COMT inhibitors according to their central bioavailability and pharmacokinetics, as well as between diseases depending on the nature and severity of dopaminergic involvement in their pathophysiology (4,59–62).

## Figures and Tables

Idea from Tunbridge *et al.* (7) and Mattay *et al.* (22).

**Table 1:** Demographics of Subjects

	Met-COMT, Placebo	Val-COMT, Placebo	Met-COMT, Placebo	Met-COMT, Placebo	ANOVA, *p*
Number^a^	18	16	16	17	
Ethnicity^b^	17C, 1I	12C, 2I, 1Ch, 1A	16C	14C, 2I, 1Ch	
Age (Years)	22.6 (3.2)	24.0 (5.1)	24.4 (9.0)	23.9 (3.9)	.80
NART	118 (4)	117 (5)	115 (5)	116 (6)	.23
Alcohol (Units/Week)	10 (7)	8 (5)	7 (5)	8 (8)	.59
Cigarettes/Day	0 (0)	.4 (1.3)	.4 (1.5)	.2 (.7)	.60
BDI	3.9 (5.3)	3.5 (3.3)	3.7 (3.7)	3.1 (2.9)	.89
STAI	32 (8)	39 (7)	33 (10)	37 (8)	.09

Values are mean (SD). ANOVA, analysis of variance; BDI, Beck Depression Inventory; COMT, catechol-*O*-methyltransferase Val^158^Met genotype; NART, National Adult Reading Test; STAI, Spielberger Trait Anxiety Inventory.

a Seven subjects completed the gambling task but not the N-back. Final sample size for the N-back task was as follows: *n* = 15 (Met-COMT placebo), 15 (Val-COMT placebo), 16 (Met-COMT tolcapone), and 14 (Val-COMT tolcapone). The demographics of the 60 who completed the N-back were virtually identical to those given here for the whole sample.

b C, Caucasian; Ch, Chinese; I, Indian; A, African Caribbean.

**Table 2:** Reaction Times and Additional Gambling Task Data

	Met-COMT, Placebo	Val-COMT, Placebo	Met-COMT, Tolcapone	Val-COMT, Tolcapone
N-Back Task^a^ (msec)				
0-Back RT	504 (99)	494 (68)	531 (104)	488 (65)
1-Back RT	540 (199)	627 (200)	608 (200)	497 (172)
2-Back RT	653 (237)	844 (256)	693 (233)	687 (282)
3-Back RT	706 (290)	916 (269)	690 (255)	707 (245)
Gambling Task^b^				
RT (msec)	730 (333)	715 (331)	884 (557)	667 (187)
Choose 5 after 2 gains^c^ (%)	57 (21)	50 (17)	47 (19)	63 (18)
Choose 5 after 2 losses^d^ (%)	45 (22)	34 (15)	38 (16)	50 (18)

Values are mean (SD). COMT, catechol-*O*-methyltransferase Val^158^Met genotype; RT, reaction time.

a RT not available for three subjects. For statistics, see text.

b RT not available for 10 subjects.

c Genotype × drug interaction [*F*(1,61) = 5.96, *p =* .018]. For subjects on tolcapone, Val-COMT > Met-COMT, *p =* .036.

d Genotype × drug interaction [*F*(1,61) = 6.65, *p =* .012]. For subjects on tolcapone, Val-COMT > Met-COMT, *p =* .023. For Val-COMT, tolcapone > placebo, *p =* .043.
