# *COMT* Val^158^Met Genotype Determines the Direction of Cognitive Effects Produced by Catechol-*O*-Methyltransferase Inhibition

**DOI:** 10.1016/j.biopsych.2014.12.004

**Published:** 2015-02-01

An error in Table 1 has been discovered in the article “*COMT* Val^158^Met Genotype Determines the Direction of Cognitive Effects Produced by Catechol-*O*-Methyltransferase Inhibition” by Farrell *et al.* (2012;71:538-544). Specifically, in Table 1 (page 539), the 3rd and 4th column headings are incorrect. The 3rd column heading should read “Met-COMT, Tolcapone” and the 4th column heading should read “Val-COMT, Tolcapone”. The data in the table are correct and remain unchanged. The table with the corrected headings is reprinted here in its entirety.

**Table 1:** Demographics of Subjects

	**Met-COMT, Placebo**	**Val-COMT, Placebo**	**Met-COMT, Tolcapone**	**Val-COMT, Tolcapone**	**ANOVA,*****p***
Number^a^	18	16	16	17	
Ethnicity^b^	17C, 1I	12C, 2I, 1Ch, 1A	16C	14C, 2I, 1Ch	
Age (Years)	22.6 (3.2)	24.0 (5.1)	24.4 (9.0)	23.9 (3.9)	.80
NART	118 (4)	117 (5)	115 (5)	116 (6)	.23
Alcohol (Units/Week)	10 (7)	8 (5)	7 (5)	8 (8)	.59
Cigarettes/Day	0 (0)	.4 (1.3)	.4 (1.5)	.2 (.7)	.60
BDI	3.9 (5.3)	3.5 (3.3)	3.7 (3.7)	3.1 (2.9)	.89
STAI	32 (8)	39 (7)	33 (10)	37 (8)	.09

Values are mean (SD).

ANOVA, analysis of variance; BDI, Beck Depression Inventory; COMT, catechol-*O*-methyltransferase Val^158^Met genotype; NART, National Adult Reading Test; STAI, Spielberger Trait Anxiety Inventory.

a Seven subjects completed the gambling task but not the N-back. Final sample size for the N-back task was as follows: *n* = 15 (Met-COMT placebo), 15 (Val-COMT placebo), 16 (Met-COMT tolcapone), and 14 (Val-COMT tolcapone). The demographics of the 60 who completed the N-back were virtually identical to those given here for the whole sample.

b C, Caucasian; Ch, Chinese; I, Indian; A, African Caribbean.

