# Supplementary material for: COMT Val158Met Genotype Determines the Direction of Cognitive Effects Produced by Catechol-O-Methyltransferase Inhibition
Source: Biol Psychiatry. 2012 Mar 15;71(6):538–44. doi: 10.1016/j.biopsych.2011.12.023 (PMC3314969; doi:10.1016/j.biopsych.2011.12.023)
Supplement: Supplement 1 [file mmc1.pdf]

COMT Val^{158}Met Genotype Determines the Direction of Cognitive Effects Produced by Catechol-*O*-Methyltransferase Inhibition

Supplemental Information

Table S1. Visual Analogue Scale scores

		T1	T2	T3
Alertness	Met Placebo	6.8 (1.6)	7.0 (2.3)	6.3 (2.3)
	Val Placebo	6.0 (2.1)	5.9 (2.1)	5.8 (2.6)
	Met Tolcapone	5.9 (2.2)	5.8 (2.0)	5.1 (2.4)
	Val Tolcapone	6.0 (1.6)	6.2 (2.0)	5.5 (2.0)
	All Met	6.3 (1.9)		
	All Val	6.0 (1.8)		
Drowsiness	Met Placebo	2.2 (2.2)	2.6 (2.6)	3.0 (2.9)
	Val Placebo	3.7 (3.0)	3.4 (2.3)	2.9 (2.7)
	Met Tolcapone	3.7 (2.6)	3.1 (2.5)	4.0 (2.3)
	Val Tolcapone	3.4 (2.0)	2.8 (1.9)	3.5 (2.0)
	All Met	2.9 (2.5)		
	All Val	3.6 (2.5)		
Anxiety	Met Placebo	1.1 (1.0)	0.9 (1.1)	0.5 (0.6)
	Val Placebo	2.0 (1.8)	1.5 (1.6)	1.4 (1.8)
	Met Tolcapone	2.0 (1.8)	1.6 (1.3)	1.5 (1.7)
	Val Tolcapone	1.2 (0.8)	1.2 (1.4)	1.0 (1.1)
	All Met	1.5 (1.5)		
	All Val	1.6 (1.4)		
Happiness	Met Placebo	6.9 (1.5)	7.5 (1.3)	7.1 (1.7)
	Val Placebo	6.1 (1.7)	6.4 (1.6)	5.9 (1.9)
	Met Tolcapone	7.3 (1.6)	7.2 (1.6)	7.1 (2.3)
	Val Tolcapone	5.9 (2.0)	6.4 (1.5)	6.9 (1.4)
	All Met	7.1 (1.6)		
	All Val	6.0 (1.8)		
Sadness	Met Placebo	0.7 (0.8)	0.6 (0.8)	0.3 (0.4)
	Val Placebo	1.2 (1.2)	1.0 (1.7)	1.0 (1.8)
	Met Tolcapone	1.0 (1.3)	0.9 (1.0)	1.2 (1.3)
	Val Tolcapone	1.6 (1.7)	1.6 (2.0)	1.7 (1.9)
	All Met	0.9 (1.1)		
	All Val	1.4 (1.5)		
Nausea	Met Placebo	0.6 (1.8)	0.7 (1.8)	0.5 (1.2)
	Val Placebo	0.9 (1.6)	0.8 (1.8)	0.8 (2.0)
	Met Tolcapone	0.9 (1.2)	0.7 (1.2)	1.0 (1.5)
	Val Tolcapone	0.9 (1.8)	0.6 (0.8)	0.8 (1.3)
	All Met	0.8 (1.5)		
	All Val	0.9 (1.6)		

T1: ratings taken just before the subjects received the tolcapone or placebo capsule. T2: ~90 minutes later. T3: a further ~120 minutes later.

Table S2. Pearson correlations between N-back performance, risky choices, and happiness Visual Analogue Scale (VAS) scores.

	**N-back Accuracy**				**Gambling Task**	**Happiness VAS Score**		
	**0-back**	**1-back**	**2-back**	**3-back**	**'5 not 25'**	**T1**	**T2**	**T3**
N-back Accuracy								
0-back		0.45*	0.40*	0.34*	0.05	-0.03	0.03	-0.15
1-back			0.79*	0.65*	-0.06	0.08	0.03	-0.13
2-back				0.84*	-0.09	0.14	0.12	0.04
3-back					-0.15	0.12	0.09	0.04
Gambling Task								
'5 not 25'						-0.05	0.15	0.23
Happiness VAS Score								
T1							0.79*	0.62*
T2								0.73*

Values are Pearson R correlation coefficients.
*$p < 0.005$, all other correlations non-significant.

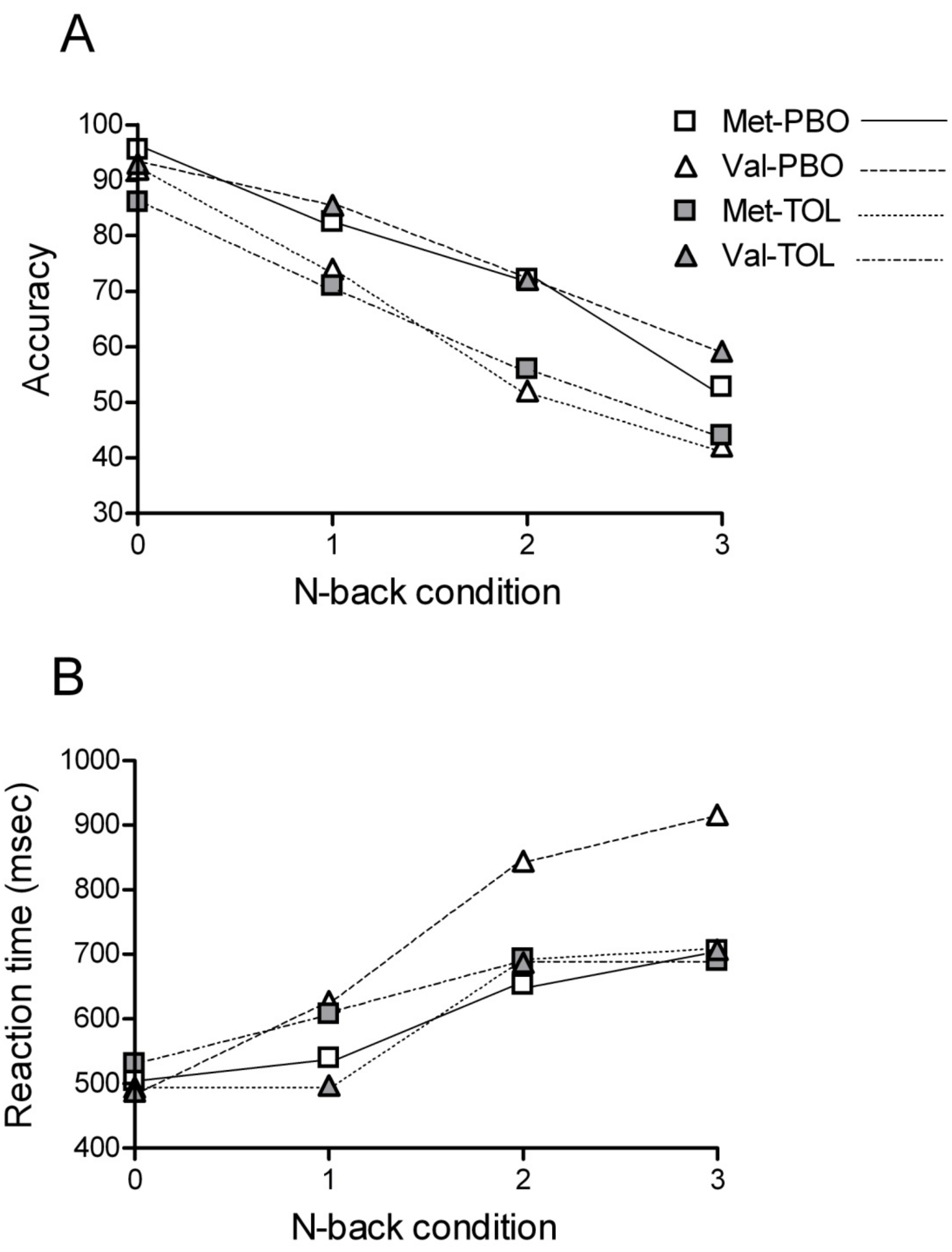

Figure S1. Accuracy and reaction times for the 0, 1, 2 and 3-back. PBO, placebo; TOL, tolcapone.
